# Researches on the Epithelium of the Cornea

**Published:** 1883-02

**Authors:** E. G. Betty

**Affiliations:** Cincinnati


### CORRESPONDENCE.

### Researches on the Epithelium of the Cornea.

Editor REGISTER; *Sir*:—The *Medical News*, of Nov. 11, 1882, contains a paper entitled "Researches on the Epithelium of the Cornea," by Dr. Eric E. Sattler, M. D., of this city. Dr. Sattler is a young man of great promise in his profession, having made his *debut* as interne at the Cincinnati Hospital from the Miami Medical College, where he graduated last spring. He is now in Europe where he is engaged in study, his experiments being made in the laboratory of Professor Waldeyer, at Strassburg.

As many readers of the REGISTER are engaged in microscopical studies, and as our author says: "The Cornea and its Epithelium have played for a long time one of the most important *roles* in the history of microscopy, and still serve as excellent objects for experimentation and generalization," a brief abstract of his researches will be of great value to them. Silver salts solution and the solid stick have been used for some time past in coloring the corneal tissues, some using the stick for observing inflammatory processes in those structures. The merit belongs to Dr. Sattler for employing it "in clearly demonstrating the *structure* of the epithelium." The mode of procedure is as follows: after removing the membrana nictitaus of a recently killed frog, the eye is slightly pressed from the orbit, with the finger introduced into the jaw, and the solid stick freely applied to the surface of the cornea until it has assumed a milky appearance. The pure nitrate stick is greatly to be preferred to the mitigated. The upper part of the head with the eyes still remaining in the orbits, is then removed from the rest of the body, and is exposed in slightly acidulated water (formic or acetic acid) to the direct rays of the sun. This latter procedure is not absolutely necessary; as good, if not better, results may be obtained by simple exposure of the head to the sun, without the addition of the acidulated water. As soon as the corneæ have assumed a brownish color, they are carefully excised in the usual way, spread out with glycerine under a cover-glass, and looked at with a high power. 'On sunny days, fifteen to thirty minutes are all that is required to complete the whole procedure.' The method of staining is given in detail for those who wish to experiment. The doctor then goes on to describe the appearances of the cells, neuclei, and neucleoli under the microscope. The advantage of using the solid stick is, that the neucleoli are distinctly visible with the one staining; other methods requiring a second application of the coloring matter. 'The silver method constantly shows the appearance of the entire neuclous with its chromatic and achromatic substance, which, however, cannot be distinguished from one another.' To fully demonstrate the process of division taking place in the neucleus there is need of further investigation, new methods of staining may be discovered, that will fully demonstrate much that is now hypothetical. The author has seen and been able to trace several instances of the division of the neucleus into three new ones. Another advantage of the silver staining is the facility it offers for exact measurement of the cells. He also noticed constantly recurring, fine, black lines apparently originating in the intercellular cement substance and ending in the neuclei; these he suggests may be nerves, though he is unwilling to make a hasty statement upon insufficient data." E. G. BETTY, D. D. S.

Cincinnati, January 25, 1883.

